# Electroconvulsive Therapy in Treatment-Resistant Depression: A Comprehensive Literature Review

**DOI:** 10.1192/j.eurpsy.2026.11403

**Published:** 2026-07-31

**Authors:** A. Rami

**Affiliations:** Razi Hospital, Manouba, Tunisia

## Abstract

**Introduction:**

Treatment-resistant depression (TRD) affects approximately one-third of patients with major depressive disorder and remains a significant public health concern due to its chronicity, disability, and high suicide risk. Among available therapeutic strategies, electroconvulsive therapy (ECT) — also known as electroconvulsive seizure therapy or sismotherapy — continues to demonstrate robust efficacy, particularly in severe or pharmacologically refractory cases.

**Objectives:**

This literature review aims to synthesize contemporary evidence regarding the efficacy, neurobiological mechanisms, safety, and clinical positioning of ECT in the management of treatment-resistant depression.

**Methods:**

A narrative review of articles published between 2010 and 2025 was conducted using PubMed, ScienceDirect, and PsycINFO. Randomized controlled trials, meta-analyses, and clinical guidelines were included to assess both therapeutic outcomes and cognitive effects associated with modern ECT protocols.

**Results:**

Across studies, ECT shows **response rates of 60–80%** and **remission rates around 50–60%**, outperforming all other interventions for TRD. Advances in technique, such as **unilateral ultrabrief pulse stimulation**, **optimized seizure monitoring**, and **individualized titration**, have significantly improved tolerability while preserving efficacy. Neurobiological findings implicate **modulation of fronto-limbic circuits**, **enhanced neuroplasticity**, and **upregulation of BDNF** as key mechanisms underlying clinical response. The most frequent adverse effects remain **transient cognitive disturbances**, mainly short-term memory deficits, which tend to resolve within weeks.

**Conclusions:**

ECT remains the **most effective and rapidly acting intervention** for severe, treatment-resistant, and suicidal depression. Its modern refinements have greatly enhanced safety and reduced cognitive burden. Despite persistent stigma and underutilization, ECT should be considered a **first-line option for TRD**, especially in cases of life-threatening depression or when rapid remission is critical. Continued research on biomarkers and maintenance strategies may further optimize its clinical integration.

**Disclosure of Interest:**

None Declared

